# Supplementary material for: Facile accelerated specific therapeutic (FAST) platform develops antisense therapies to counter multidrug-resistant bacteria
Source: Commun Biol. 2021 Mar 12;4:331. doi: 10.1038/s42003-021-01856-1 (PMC7955031; doi:10.1038/s42003-021-01856-1)
Supplement: Supplementary file 2 — Supplementary Information [file 42003_2021_1856_MOESM2_ESM.pdf]

Supplementary information for:

Facile Accelerated Specific Therapeutic (FAST) Platform Develops Peptide Nucleic Acid Therapies to Counter Multidrug-Resistant Bacteria

Kristen A. Eller[1], Thomas R. Aunins[1], Colleen M. Courtney[1,4], Jocelyn K. Campos[1], Peter B. Otoupal[1], Keesha E. Erickson[1], Nancy E. Madinger[2], and Anushree Chatterjee[1,3,4,5*]

[1]Chemical and Biological Engineering, University of Colorado Boulder, Boulder, CO, USA 80303

[2]Division of Infectious Diseases, University of Colorado Denver, Aurora, CO, USA 80045

[3]Biomedical Engineering, University of Colorado Boulder, Boulder, CO, USA 80303

[4]Sachi Bioworks, Inc, Boulder, CO, USA 80301

[5]Antimicrobial Regeneration Consortium, Boulder, CO, USA 80301

[*]Corresponding Author: Email: chatterjee@colorado.edu

Supplementary Discussion

Single basepair mismatch off-targets: α-rpsD in *S. enterica* showed homology with the start site of *rtcA* (Table S5) when allowing for zero basepair mismatches. The *rtcA* gene codes for RNA 3'-terminal phosphate cyclase and plays a role in end healing within an RNA repair pathway[1]. When allowing for a 1-bp mismatch on the start codon for all the PNAs, we found no off-targets for α-lexA, α-gyrB, α-fnr, and α-recA whereas α-ffh, α-csgD, α-folC, α-acrA, α-rpsD showed 2, 2, 3, 3, 5 off-targets respectively (Fig. S6A-D, Table S5). A single basepair-mismatch does not necessarily prevent binding to the corresponding RNA sequence but will significantly lower efficiency and increase the minimum inhibitory concentration (MIC)[2]. Using an electrophoretic mobility gel shift assay (EMSA) we find that both α-rpsD (five 1-bp mismatches) and α-lexA (zero 1-bp mismatches) show binding specificity to their target (Fig. S6E, Table S6).

Antibiotic resistant genome screening of clinical isolates: CRE *E. coli* was found to have five β-lactam resistance genes, two aminoglycoside resistance genes, and one gene each for phenicol, tetracycline, sulfonamide, and trimethoprim resistance. MDR *E. coli* was found to have six β-lactam resistance genes, one aminoglycoside resistance gene, and one gene each for phenicol and trimethoprim resistance. ESBL KPN was found to have seven β-lactam resistance genes, two aminoglycoside resistance genes, and one fluoroquinolone resistance gene. NDM-1 KPN was found to have seven β-lactam resistance genes, three genes each for fluoroquinolone and aminoglycoside resistance and one gene each for tetracycline, sulfonamide, and trimethoprim resistance. MDR STm was found to have one β-lactam resistance gene and one aminoglycoside resistance gene. The specific resistance genes are noted in Table S3.

Predicted RNA Folding of PNA targets: The folding of each PNA target theoretical sequence was analyzed using the RNAfold Web[3], looking at about 50 basepairs up and downstream of the target site. The PNA target regions of *folC* show relatively high local positional entropy, while the *rpsD*, *lexA*, and *csgD* target regions exhibit low entropy (Fig. S9). Despite this, α-folC, α-rpsD α-lexA, and α-csgD PNAs showed varying degree of effectiveness against the MDR isolates, suggesting that RNA secondary structure has little influence on PNA efficacy.

Protein network interactions affected by knockdown PNA targets: Based on protein network interactions predictions from the STRING Database[4] (Fig. S10) Fnr, CsgD, and LexA tend to interact with few other proteins. Cluster coefficients indicate that these interact with very specific types of proteins that often also interact with one another. RpsD and Ffh exhibited the highest levels of nodes and average node degree. PNA targeting of *rpsD* and *ffh* are predicted to therefore result in the greatest level of cascading effects throughout dissimilar pathways in the cell and could explain the PNA's relative high success as monotherapies.

Supplemental Figures

PNA	Target Sequence (5'→3')	PNA Sequence (N→C terminus)	Essentiality	Homology		
				E. Coli	KPN	STm
α-folC	ATACC**ATG**ATTA	$(KFF)_3$K-O-TAATCATGGTAT	Essential	X		
α-ffh	GACA**ATG**TTTGA	$(KFF)_3$K-O-TCAAACATTGTC	Essential	X	X	X
α-lexA	CGGA**ATG**AAAGC	$(KFF)_3$K-O-GCTTTCATTCCG	Essential	X	X	X
α-gyrB	GTTG**ATG**TCGAA	$(KFF)_3$K-O-TTCGACATCAAC	Essential	X	X	X
α-rpsD	AGAAA**ATG**GCAA	$(KFF)_3$K-O-TTGCCATTTTCT	Essential	X	X	X
α-acrA	GAGGTTTACAT**A**	$(KFF)_3$K-O-TATGTAAACCTC	Non-essential	X	X	X
α-csgD	GGGGTTTCATC**A**	$(KFF)_3$K-O-TGATGAAACCCC	Non-essential	X		
α-fnr	AGACCT**ATG**ATC	$(KFF)_3$K-O-GATCATAGGTCT	Non-essential	X		
α-recA	**ATG**GCTATCGAC	$(KFF)_3$K-O-GTCGATAGCCAT	Non-essential	X		X

Figure S1. Homology of antisense-PNA RNA-inhibitors in clinical isolates. After sequencing, PNA Finder tool was used to search for the 12 nucleotide antisense-PNA targets in the gene of interest. The PNA targets, predicted to have homology in the clinical strains based on the sequence of their respective, non-pathogenic and drug-sensitive strains, were present in all clinical isolate cases. Sequences are listed 5'to 3' with the antisense-PNA target underlined with the translation start codon in bold. Synthesized PNA sequences are listed N to C terminus starting with the CPP attached to the PNA antisense sequence by an O linker (O for AEEA). X indicates homology was found.

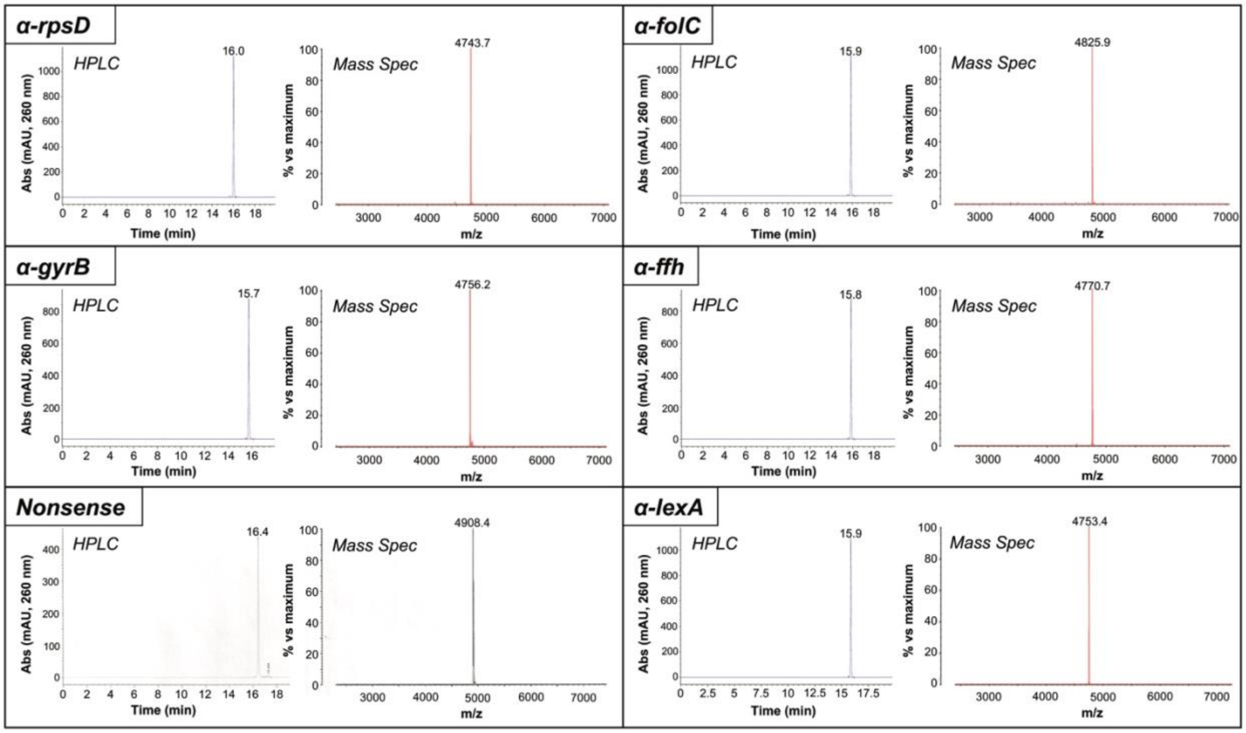

Figure S2. High performance liquid chromatography and MALDI mass spectrometry results for essential gene PNA and nonsense. HPLC was run by PNA Bio on an Agilent 1100 system at 60ºC, 1 mL/min, with a gradient of 95% water/5% acetonitrile to 80%/20% over 10 minutes, then from 80%/20% to 0%/100% over 20 minutes. MALDI MS was run by PNA Bio using an AXIMA-Assurance system (Shimadzu Biotech).

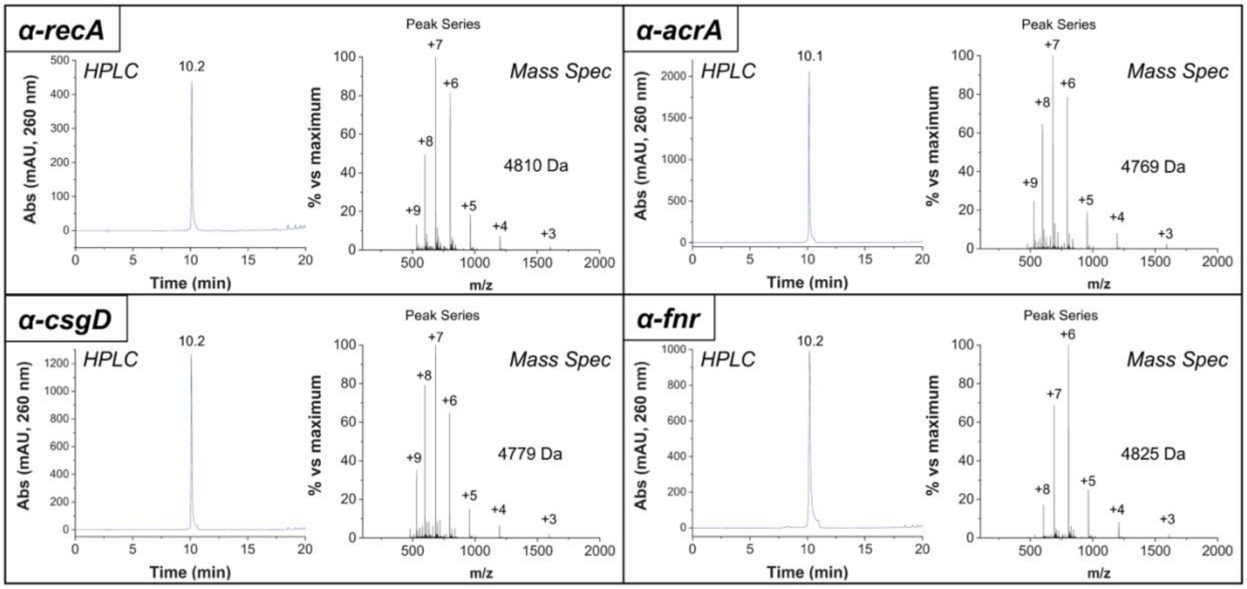

Figure S3. High performance liquid chromatography and ESI mass spectrometry results for non-essential gene PNA and nonsense. HPLC was run on an Agilent 1220 Infinity system at room temp., 1 mL/min, with a gradient of 95% water/5% acetonitrile to 5%/95% over 15 minutes. ESI MS was run under positive ion mode using a Synapt G2 HD system (Waters). Multiple charge peak series are shown as the PNA mass falls outside the instrument detection range.

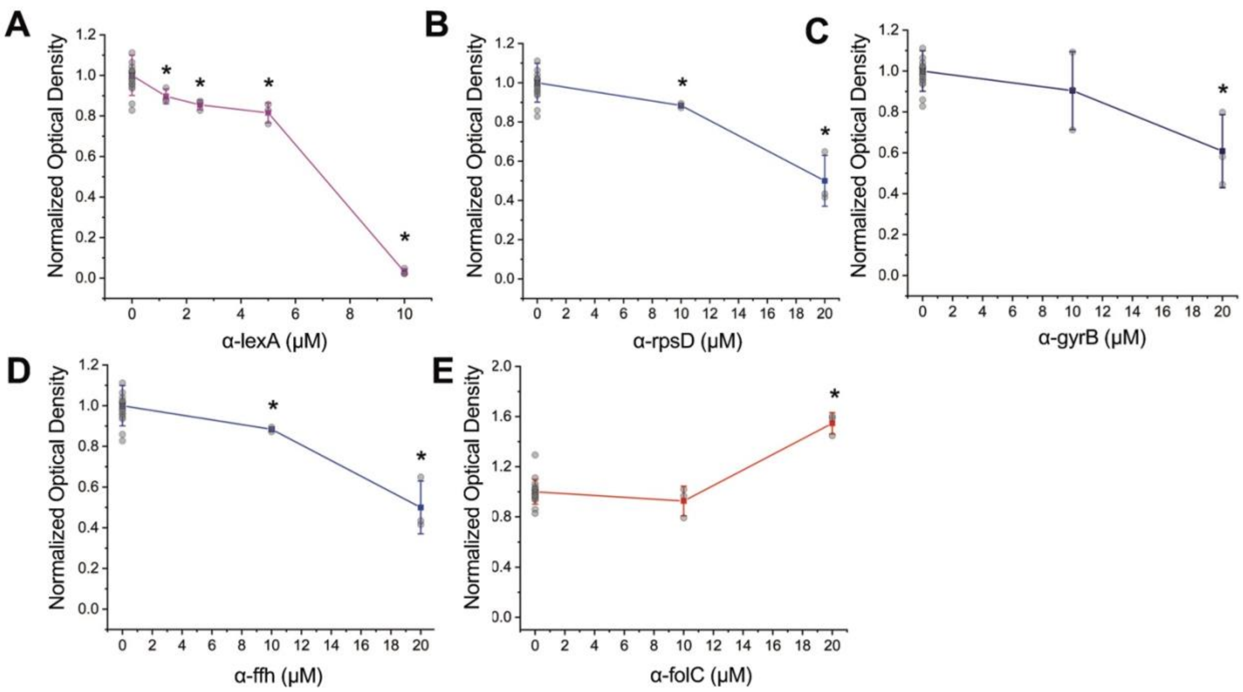

Figure S4. MG1655 dose response of various PNA. Optical density of a 1:10,000 dilution of MG6155 from overnight liquid culture, treated with various concentrations of antisense inhibitors was measured for 3 biological replicates with error bars as standard deviations. Grey circles indicate individual biological replicates. Normalized growth was measured as the optical density at 22 hours normalized to no treatment. An asterisk (*) indicates a significant difference ($p<0.05$) as compared to no treatment for each inhibitor.

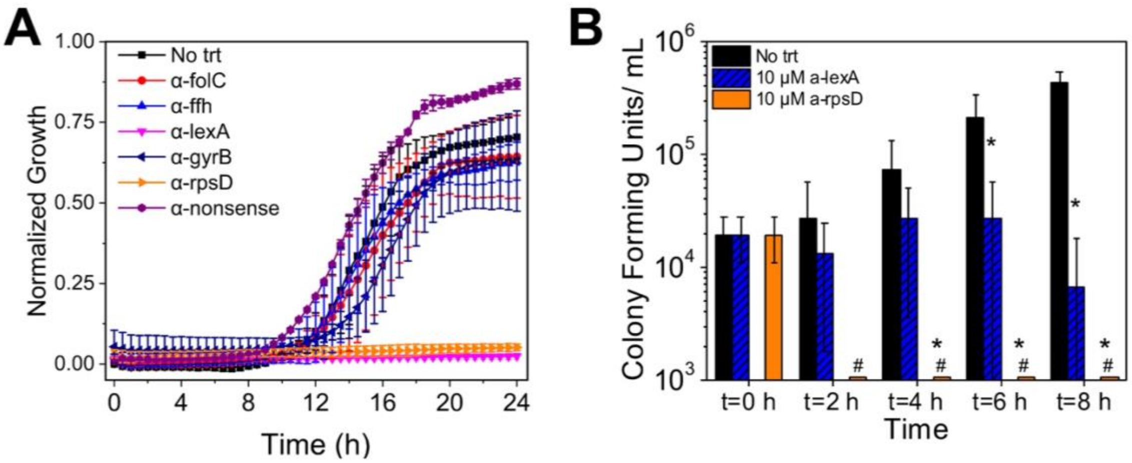

Figure S5. Growth inhibition of *E. coli* at fixed antisense concentrations. (**A**) Growth curves of *E. coli* MG1655 from a 1:10,000 dilution of an overnight culture, normalized to time t=0 with 10 µM of respective antisense inhibitor. Inhibitors α-lexA and α-rpsD show complete suppression of cell growth. (**B**) Colony forming units per milliliter of MG1655 *E. coli* for respective treatment as a function of time. The CFU/mL at t=0 represents the starting culture after a 1:100,000 dilution from overnight culture. MG1655 treatment with 10 µM α-rpsD resulted in 0 CFU/mL within 2 hours of treatment. Pound sign (#) represents significantly different from t=0 and asterisk (*) indicates significantly different from no treatment at each specified time point. Significance is measured as $p<0.05$ with n=3 biological replicates and error bars as standard deviations.

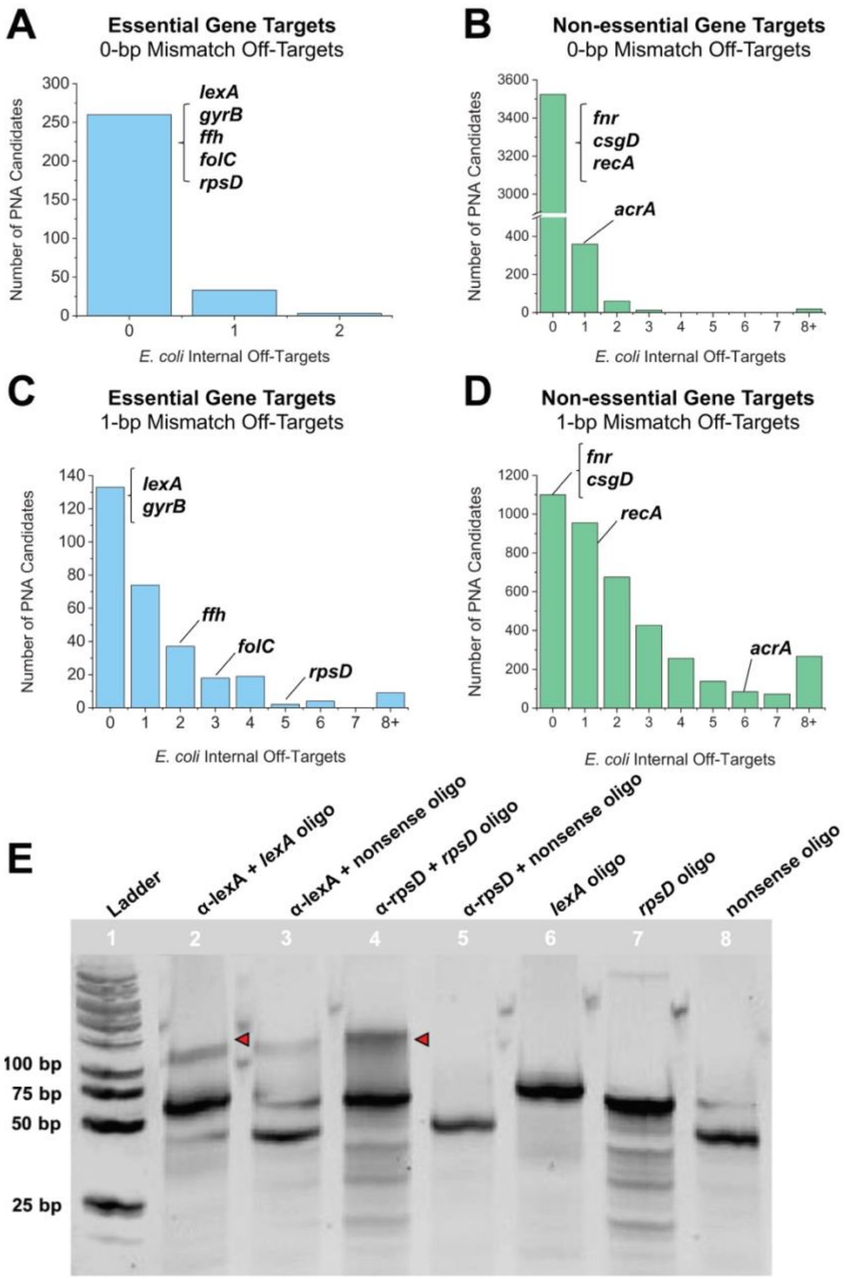

Figure S6. PNA off-target binding. (**A-D**) The set of potential PNA candidates were screened against the entire *E. coli* MG1655 genome to identify the distribution of off-target alignments when allowing for zero base mismatches in the alignment (A and B) and one base mismatch (C and D). In the 0-basepair mismatch analysis, all chosen PNAs that were tested had no other start codon alignments within the genome, except for *acrA* which had one, whereas in the one base mismatch alignment, the number of start codon alignments distribute across zero to six alignments. (**E**) Electrophoretic mobility shift

assay (EMSA) depicting 60 nucleotide single-stranded DNA fragments containing the complementation site for their corresponding PNA. Nonsense DNA was a random 60 nucleotide single-stranded DNA sequence that contained no complementation sites for PNA binding (Table S6). Lanes with only PNA showed no bands because the SYBR-Gold ® stain intercalates between the base pairs and the PNA has no secondary structure (bands not shown). Bands corresponding to PNA + complementary DNA are shifted above the unbound DNA (red triangles). Corresponding PNA + nonsense DNA bands are seen in lanes 3 and 5, with lane 3 having slight cross-over bands coming from lane 2. All lanes are from the same gel.

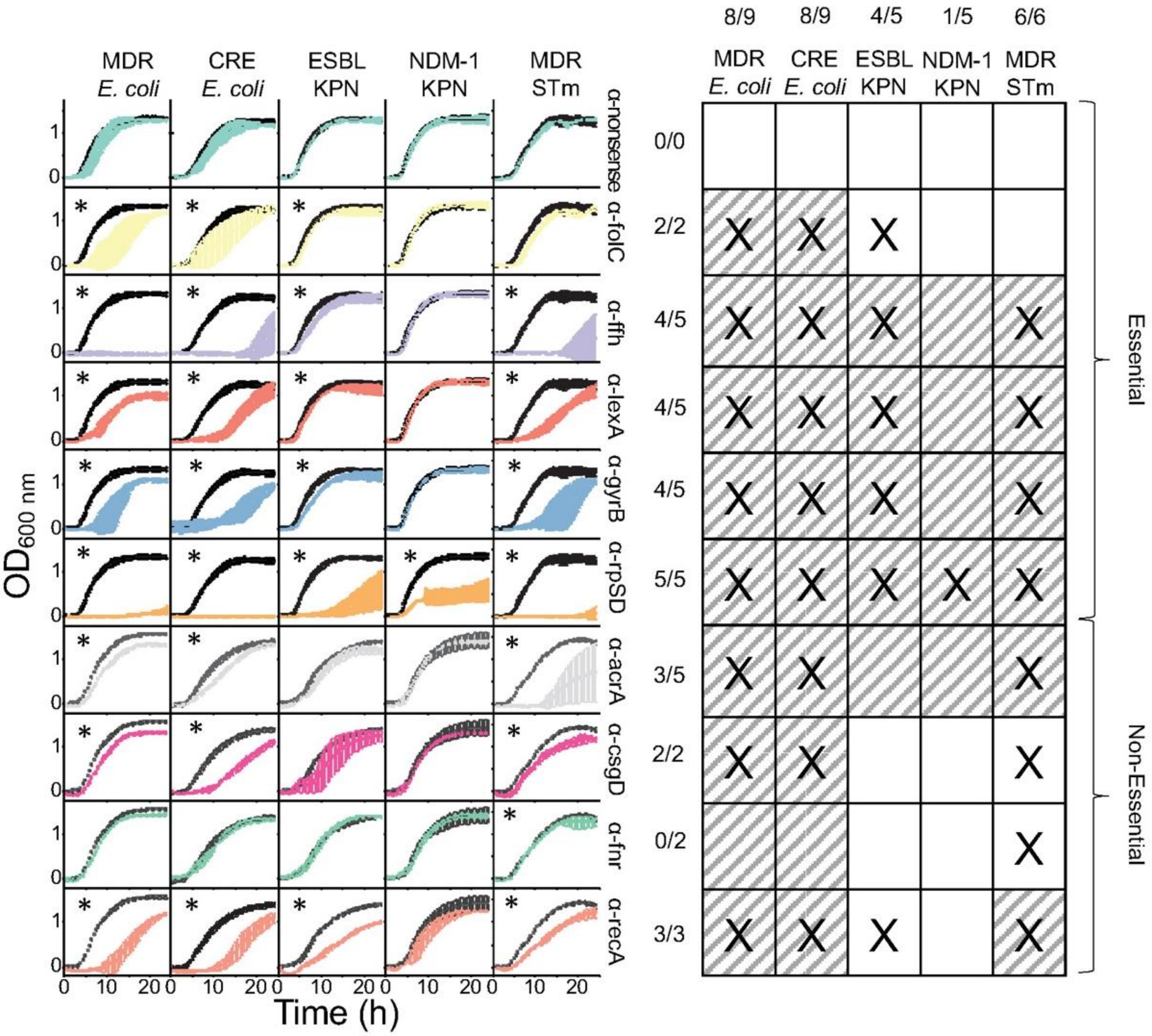

Figure S7. Growth curves of MDR clinical isolates with respective PNA monotherapy treatment. Growth curves shown are the average of at least three biological replicates normalized to the optical density at t=0 with error bars as standard deviation. Each antisense inhibitor treatment (10 µM) shown with no treatment growth curves in black for comparison. Data were used in Fig. 2D-F of the main text. An asterisk (*) indicates a significant difference ($p<0.05$) at 16 hours compared to no treatment at 16 hours. The table on the right indicates homology and efficacy of each PNA treatment. Grey diagonal lines indicate homology of the clinical isolate and PNA and an X indicates significant growth inhibition compared to no treatment. Fractions indicate the number of treatments that have homology with the isolate and which show a significant difference to

no treatment over the number of treatments that have homology, with fractions on the left corresponding to the PNA of that row and fractions above corresponding to the indicated clinical isolate.

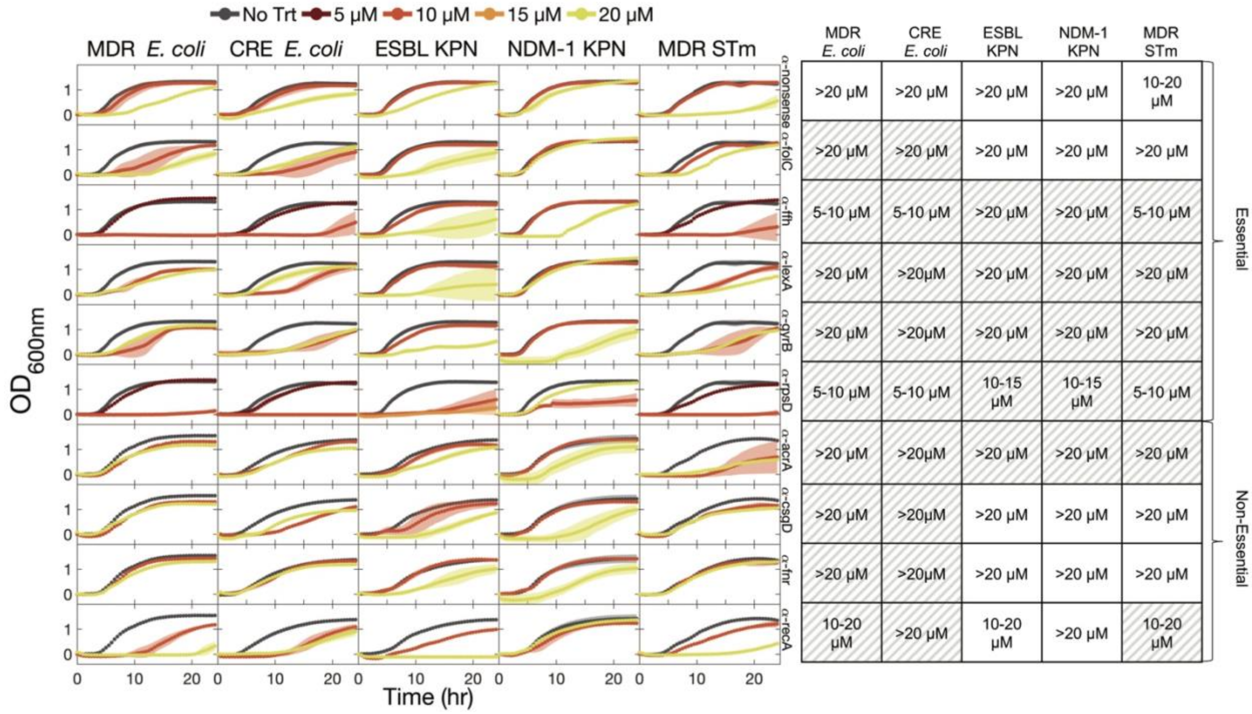

	MDR *E. coli*	CRE *E. coli*	ESBL KPN	NDM-1 KPN	MDR STm	
α-nonsense	>20 μM	>20 μM	>20 μM	>20 μM	10-20 μM	
α-folC	>20 μM	>20 μM	>20 μM	>20 μM	>20 μM	Essential
α-ffh	5-10 μM	5-10 μM	>20 μM	>20 μM	5-10 μM	Essential
α-lexA	>20 μM	>20μM	>20 μM	>20 μM	>20 μM	Essential
α-gyrB	>20 μM	>20μM	>20 μM	>20 μM	>20 μM	Essential
α-rpsD	5-10 μM	5-10 μM	10-15 μM	10-15 μM	5-10 μM	Essential
α-acrA	>20 μM	>20μM	>20 μM	>20 μM	>20 μM	Non-Essential
α-csqD	>20 μM	>20μM	>20 μM	>20 μM	>20 μM	Non-Essential
α-fnr	>20 μM	>20μM	>20 μM	>20 μM	>20 μM	Non-Essential
α-recA	10-20 μM	>20 μM	10-20 μM	>20 μM	10-20 μM	Non-Essential

Figure S8. Growth curves of MDR clinical isolates treated with varying concentrations of PNA monotherapy. Growth curves shown are the average OD_{600nm} of at least three biological replicates with time zero subtracted with error bars (standard deviation) shown as semi-transparent shaded regions. Antisense inhibitor treatment is indicated on the right side of the growth curves at varying concentrations corresponding to the colored legend. Data were used in Fig. 2G of the main text. The table on the right designates homology (grey diagonal lines) and antisense concentration ranges for 90% growth inhibition (GIC90) at 16 hours compared to no treatment.

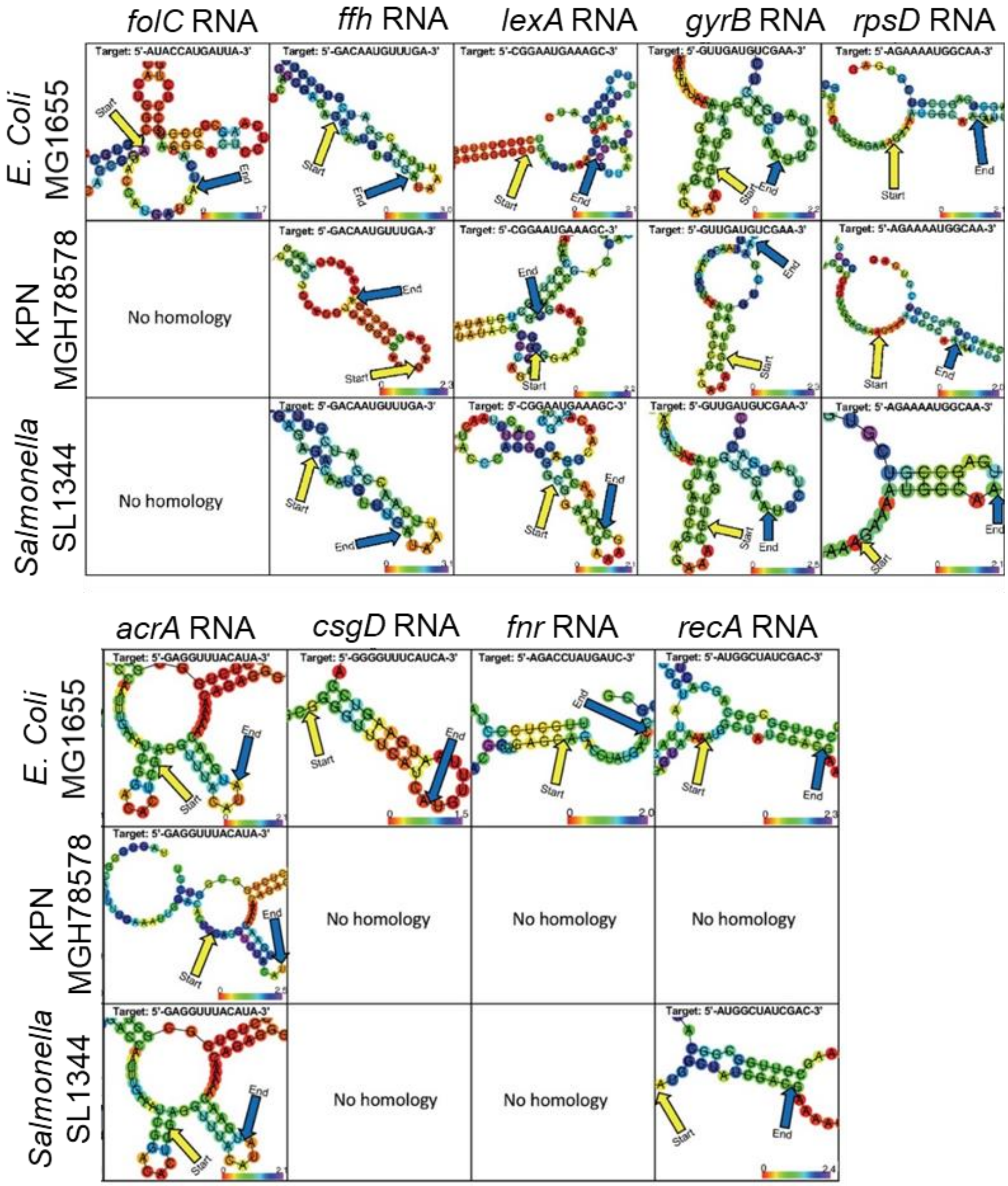

Figure S9. Predicted RNA folding of PNA-targeted mRNA. The theoretical mRNA sequences from *E. coli* MG1655, KPN MGH 8578, and Salmonella SL1344 were

analyzed using the RNAfold WebServer[3]. Folding is based on minimum free energy structures. Red shades indicate lower entropy, or areas of low conformational flexibility. The specific binding site of the PNA sequence is shown above. Top and bottom panel show essential and non-essential genes targeted in this study.

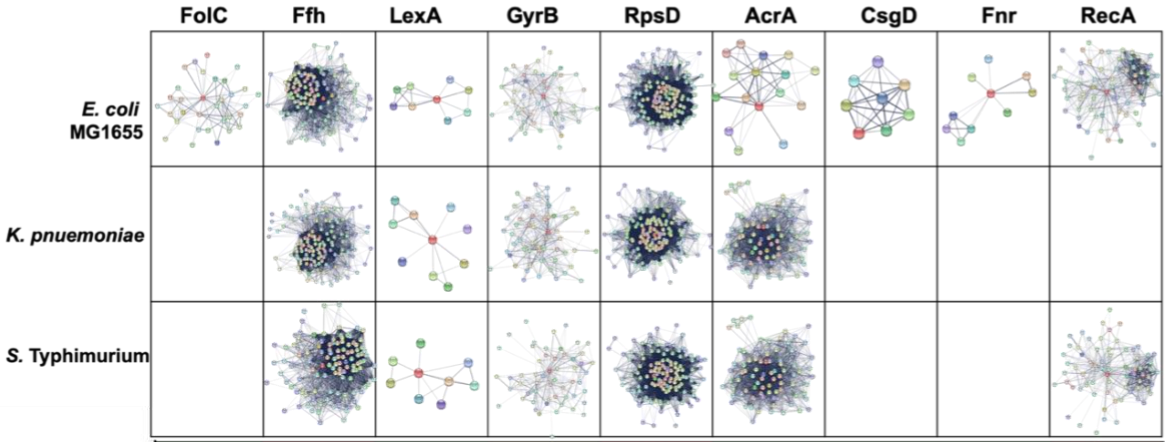

		FolC	Ffh	LexA	GyrB	RpsD	AcrA	CsgD	Fnr	RecA
Number of Nodes	*E. coli* MG1655	32	125	9	51	136	16	8	10	69
	K. pnuemoniae		139	10	56	140	82			
	S. Typhimurium		111	9	55	124	56			68
Number of Edges	*E. coli* MG1655	110	3311	17	256	5042	45	28	16	509
	K. pnuemoniae		3637	14	317	4866	1737			
	S. Typhimurium		2724	14	253	43200	775			480
Average Node Degree	*E. coli* MG1655	6.88	53	3.78	10	74.1	5.62	7	3.2	14.8
	K. pnuemoniae		52.3	2.8	11.3	69.5	42.4			
	S. Typhimurium		49.1	3.11	9.2	69.7	27.7			14.1
Average Local Clustering Coefficient	*E. coli* MG1655	0.748	0.755	0.851	0.659	0.822	0.652	1	0.919	0.652
	K. pnuemoniae		0.755	0.881	0.598	0.81	0.773			
	S. Typhimurium		0.773	0.857	0.628	0.84	0.815			0.666

Figure S10. Predicted protein interaction networks of each PNA gene target. Known protein-protein interactions (obtained from the STRING database) between the gene targeted by each PNA and other proteins throughout the cell are shown as string maps[4]. String maps present connections based on confidence, with darker lines indicating greater confidence in the existence of an interaction and node color indicating "closeness" to the target protein. The clustering coefficient representing the "tightness" of the proteins in the network. Networks were constructed for standard genomes of *E. coli* MG1655, *K. pneumoniae*, and *S.* Typhimurium. Blanks indicated where the corresponding protein targets by the PNA has no homology in the bacteria. Networks were constructed using information from the String database based on co-expression, co-occurrence, gene fusion, experimental validation, databases, and neighborhood. A medium confidence interaction score of 0.400 threshold was established and the maximum number of networks for the first shell set to 250 and the second shell set to none. Below the respective protein network interactions is a table containing various protein interaction network values such as the number of nodes, number of edges, average node degree,

and average local clustering coefficients. Each value is color coded with the lowest value of the appropriate protein network interaction value in green and the highest in red.

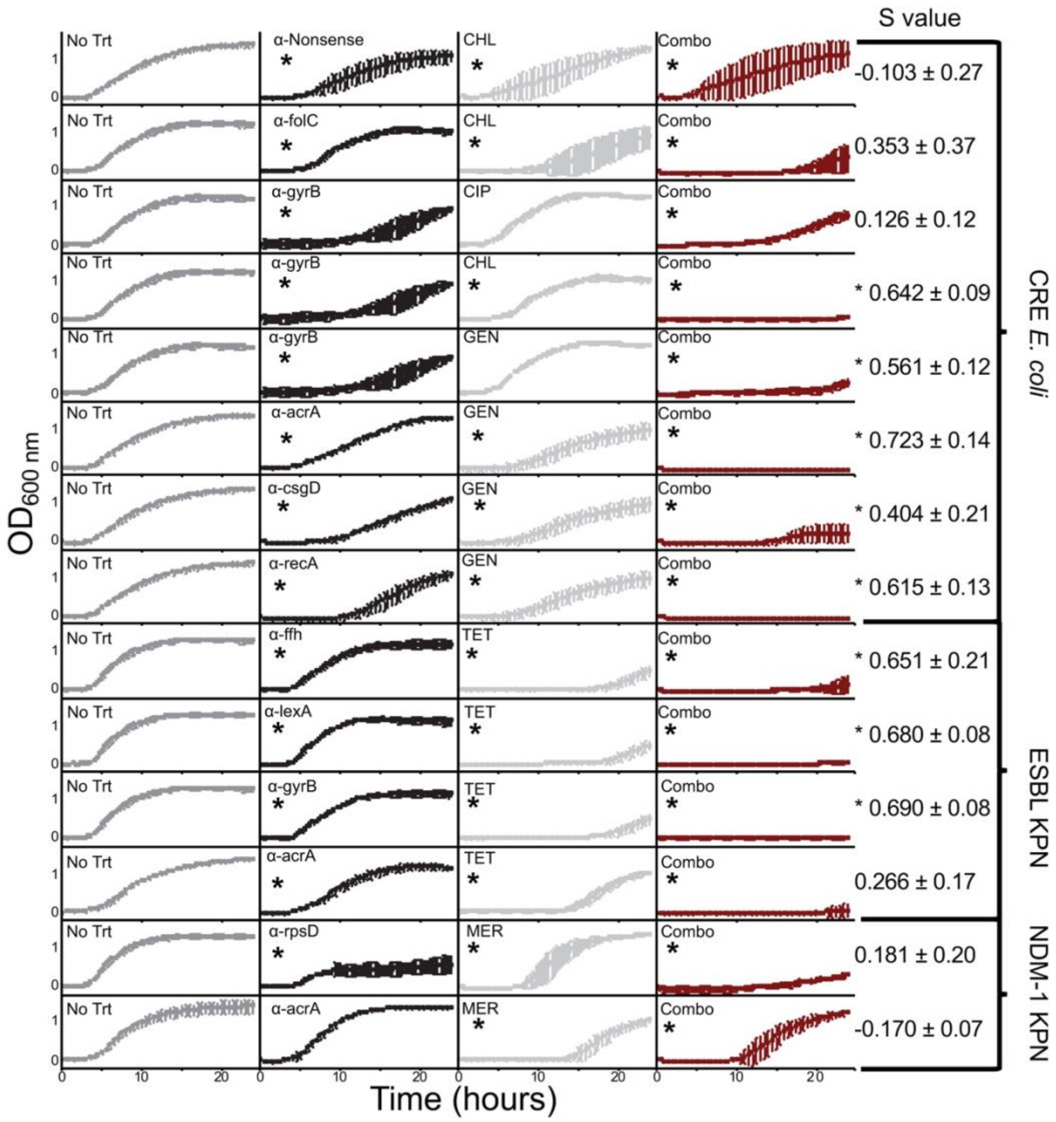

Figure S11. Growth curves of MDR clinical isolates subjected to antibiotic and PNA combination treatment. Optical density growth curves of each multidrug resistant bacteria from a 1:10,000 dilution of overnight liquid cultures, normalized to t=0. Growth curves shown are of at least three biological replicates with error bars representing standard deviation; the data shown was used in Fig. 3 of the main text. An asterisk (*) within the graph plot indicates significant inhibition ($p<0.05$) at 16 hours as compared to no treatment and an asterisk next to each S value indicates significant synergy ($p<0.05$) at 24 hours. All conditions were treated with 10 µM of the indicated PNA. Antibiotic concentrations were (from top to bottom) 8 µg/mL chloramphenicol (CHL), 1 µg/mL ciprofloxacin (CIP), 4 µg/mL gentamicin (GEN), 2 µg/mL tetracycline (TET), and 8 µg/mL meropenem (MER). CHL, CIP, and GEN are all at concentrations corresponding to their

CLSI “sensitive” breakpoints, TET is at a concentration below its CLSI “sensitive” breakpoint, and MER is at a concentration about its CLSI “resistant” breakpoint.

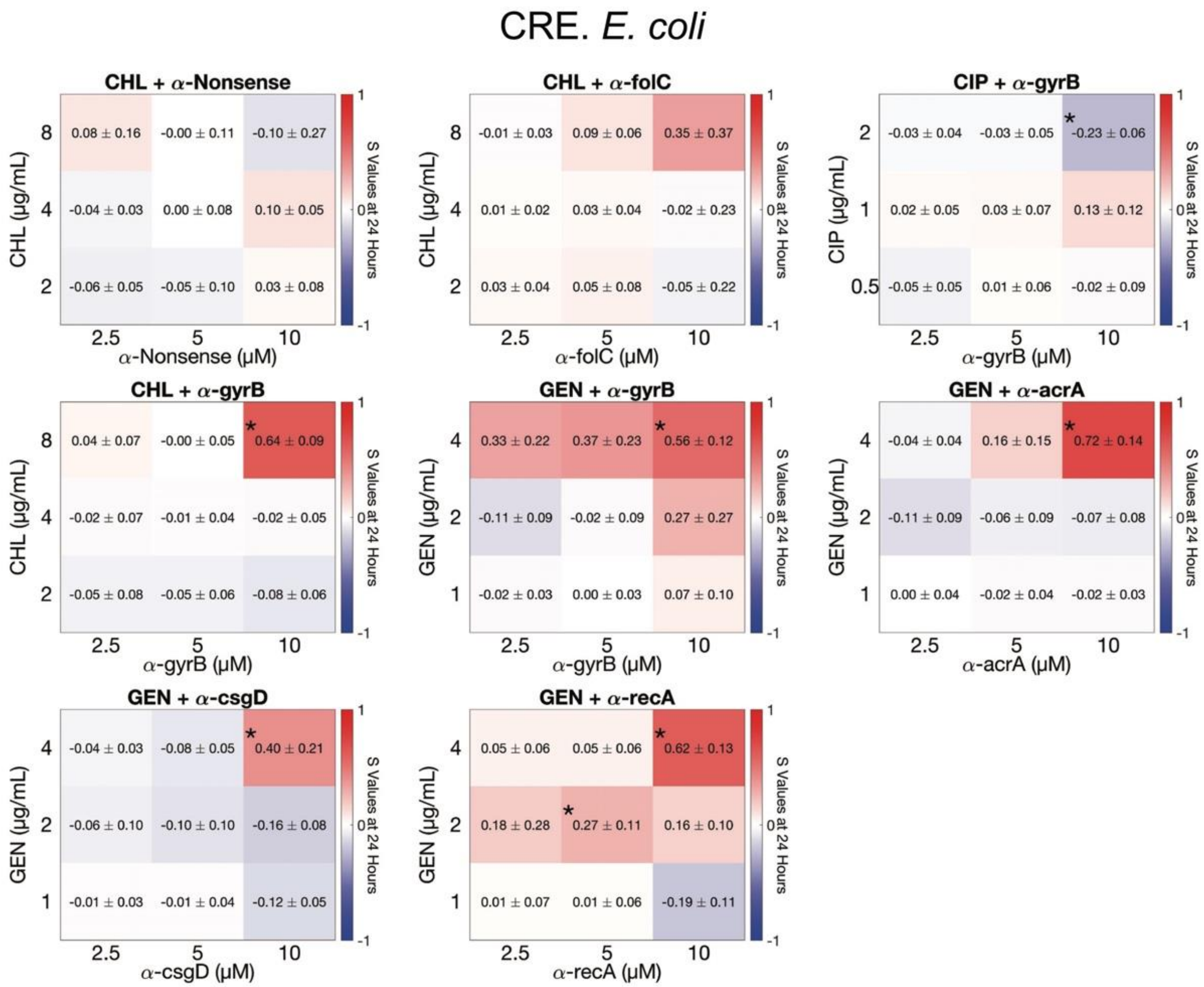

Figure S12. Heat map of antibiotic and PNA combination treatment S-values for CRE *E. coli*. Combination synergy S-values calculated using the Bliss Independence model for at least three biological replicates with error calculated as standard deviation. Heat map color scaling goes from red, synergistic interaction (S > 0), to white, no synergy (S = 0), to blue, antagonistic interaction (S < 0). An asterisk (*) indicates a statistically significant nonzero S value (p<0.05) at 24 hours.

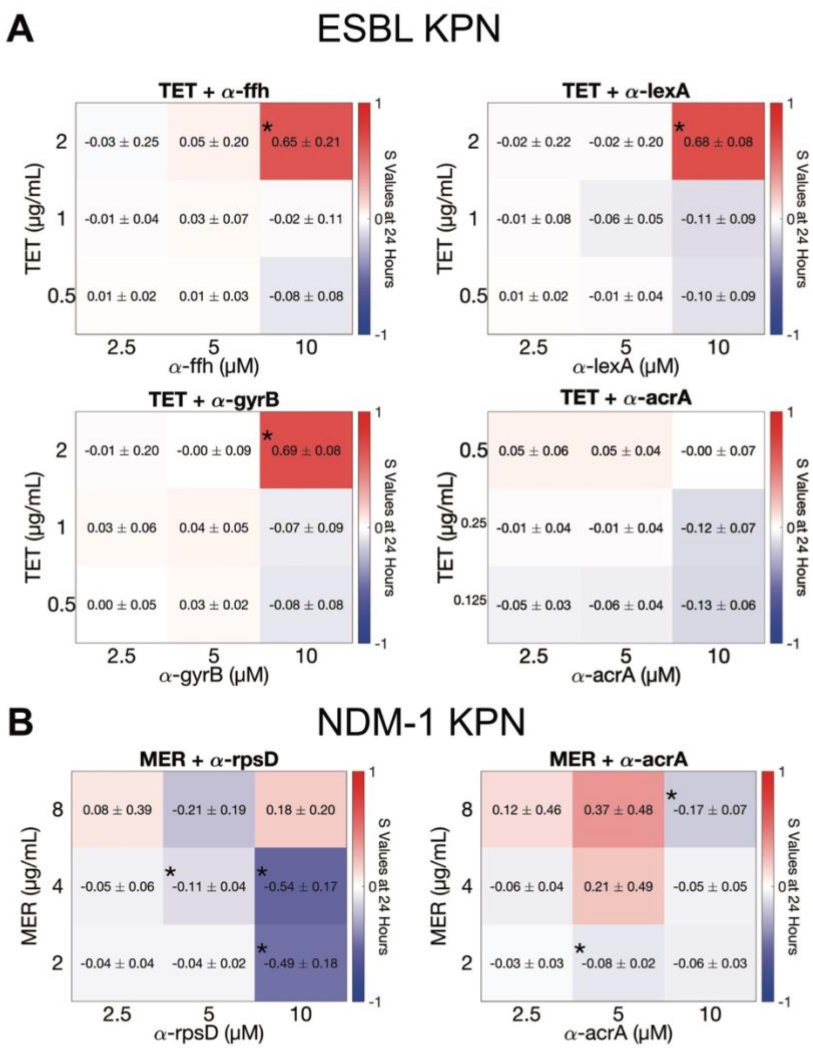

Figure S13. Heat map of antibiotic and PNA combination treatment S-values for ESBL KPN and NDM-1 KPN. Combination synergy S-values calculated using the Bliss Independence model for at least three biological replicates with error calculated as standard deviation. Heat map color scaling goes from red, synergistic interaction (S > 0), to white, no synergy (S = 0), to blue, antagonistic interaction (S < 0). An asterisk (*) indicates a statistically significant nonzero S value ($p<0.05$) at 24 hours. Tetracycline (TET) concentrations in combination with α-acrA for ESBL KPN were done at lower concentrations than previously shown (Figure S11) due to adaptation by the bacterial strain and decreased resistance to TET.

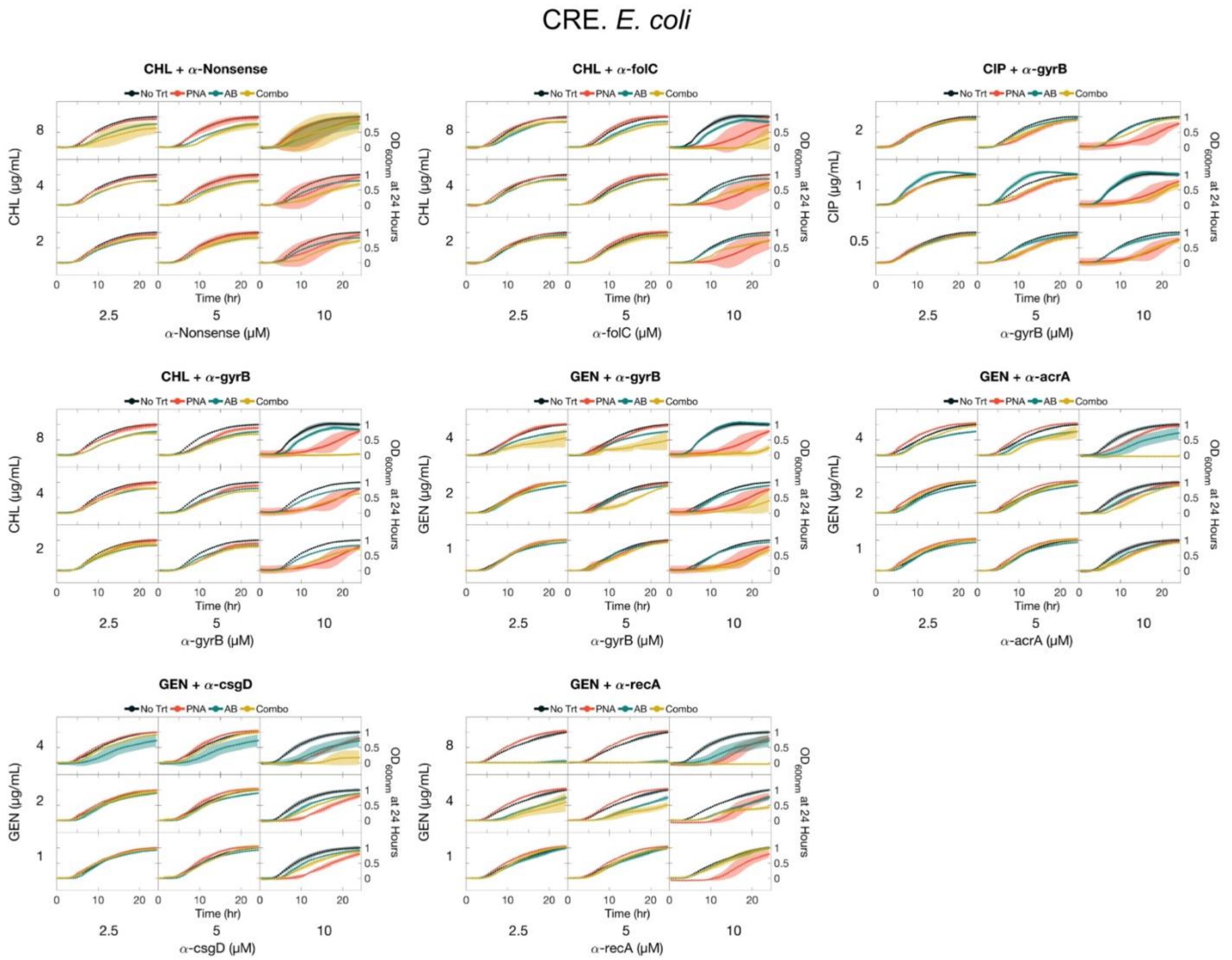

Figure S14. Growth curves of antibiotic and PNA combinations at varying concentrations for CRE *E. coli*. Optical density growth curves of combination PNA and antibiotic treatment done at varying concentrations used in Figure 3 and

S12. Growth curves are shown as OD600 nm with time zero subtracted is an average of at least three biological replicates with error bars (standard deviation) shown as semi-transparent shaded regions.

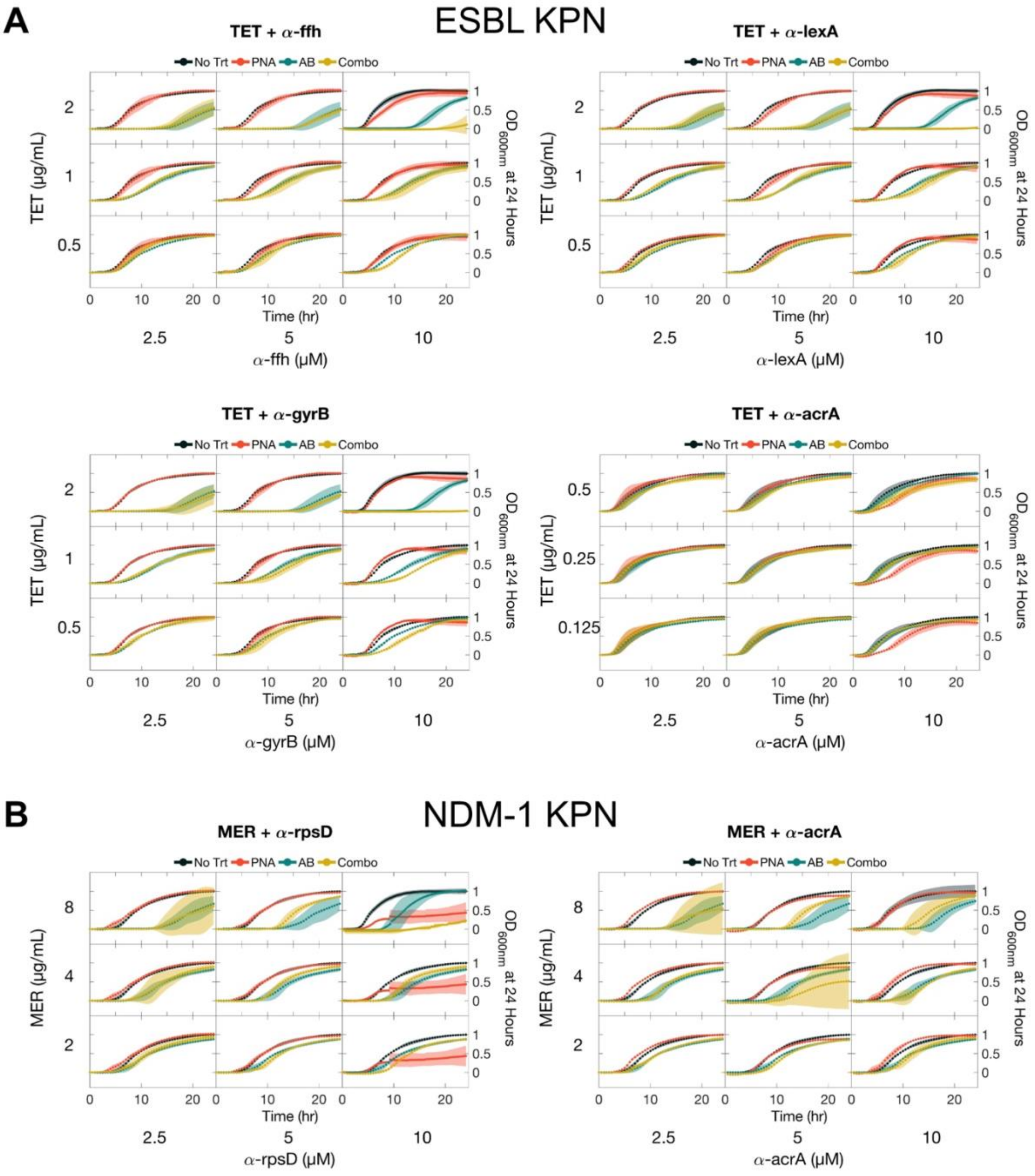

Figure S15. Growth curves of antibiotic and PNA combinations at varying concentrations for ESBL KPN and NDM-1 KPN. Optical density growth curves of combination PNA and antibiotic treatment done at varying concentrations used in Figure 3 and S13. Growth curves are shown as the average OD600 nm with time zero subtracted of at least three biological replicates and with error bars (standard deviation) shown as semi-transparent shaded regions. Tetracycline (TET) concentrations in combination with

α-acrA for ESBL KPN were done at lower concentrations than previously shown (Figure S11) due to adaptation by the bacterial strain and decreased resistance to TET.

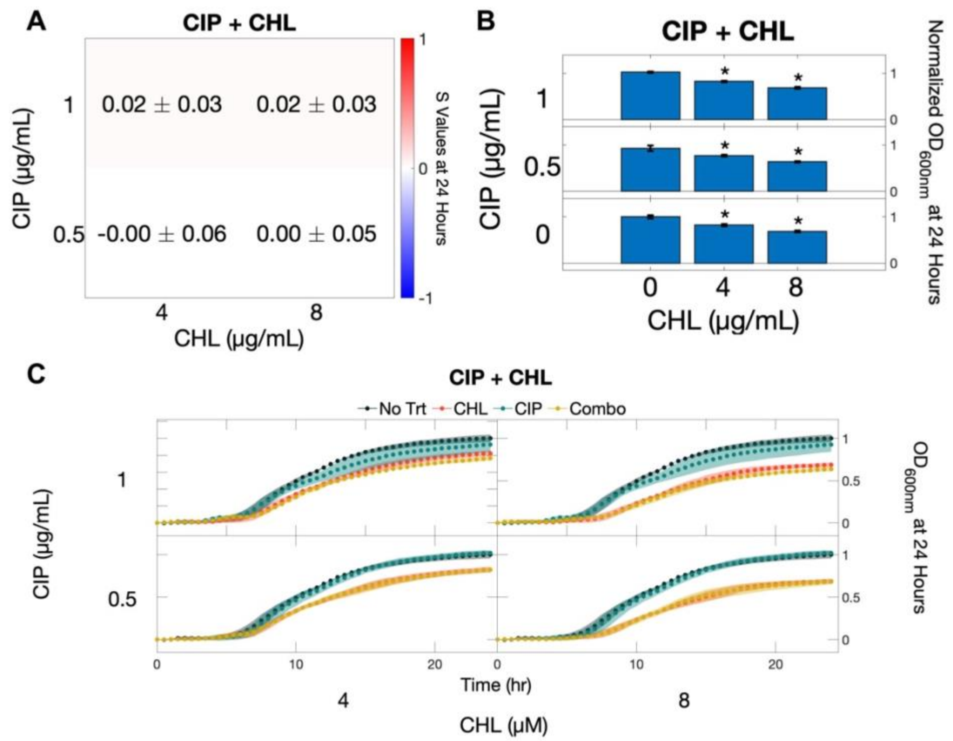

Figure S16. Evaluation of synergistic interaction between chloramphenicol and the fluoroquinolone ciprofloxacin. (**A**) Heat map of combination synergy S-values for ciprofloxacin (CIP) and chloramphenicol (CHL) interaction in ESBL KPN calculated using the Bliss Independence model. Heat map color scaling goes from red, synergistic interaction (S > 0), to white, no synergy (S = 0), to blue, antagonistic interaction (S < 0). There was no statistically significant nonzero S value ($p<0.05$) at 24 hours. (**B**) Optical density at 24 hours normalized to no treatment for each CIP and CHL combination treatment. An asterisk (*) indicates a significant difference between treatment and no treatment ($p<0.05$) at 24 hours. (**C**) Growth curves shown as optical density with time zero subtracted and with error bars (standard deviation) are shown as semi-transparent shaded regions. Averages are all of three biological replicates and error bars are standard deviation.

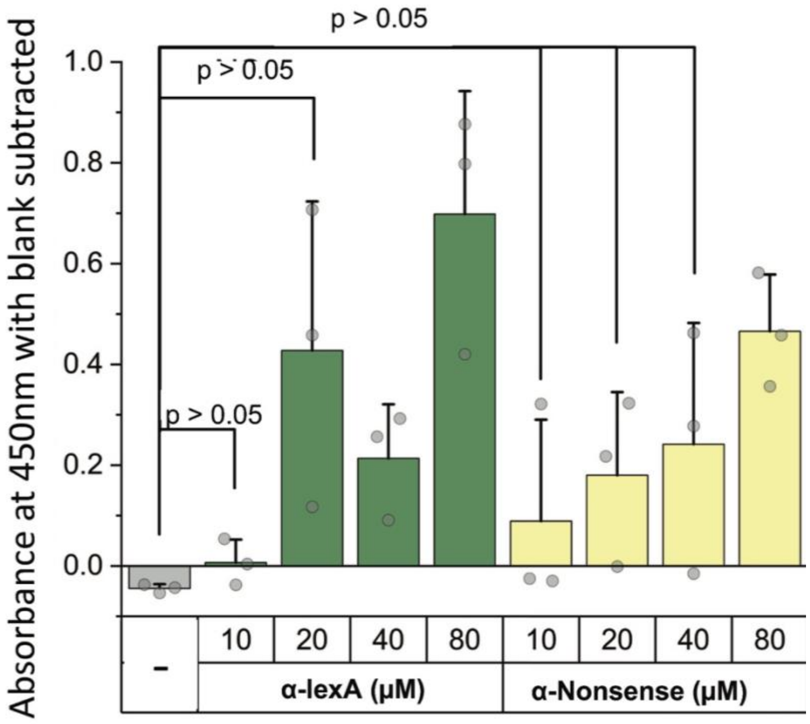

Figure S17. Cytotoxicity measurement of varying concentration of PNA in HeLa cells HeLa cells were plated on tissue culture treated 96-well plates at 4,500 cells per well. Varying concentrations of α-lexA and α-nonsense were added exogenously after 24 hours of growth and incubated in growth conditions for 18 hours. Cytotoxicity was measured using a lactate dehydrogenase assay and absorbance was read at 450 nm with a blank (growth media with lactate dehydrogenase assay) subtracted. All conditions were compared to the negative control indicated by a minus sign (-) and no significant difference, indicated by $p>0.05$, was seen for α-lexA up to 20 μM and α-nonsense up to 40 μM. Averages are of three biological replicates and error bars are standard deviations. Grey circles indicate individual biological replicates.

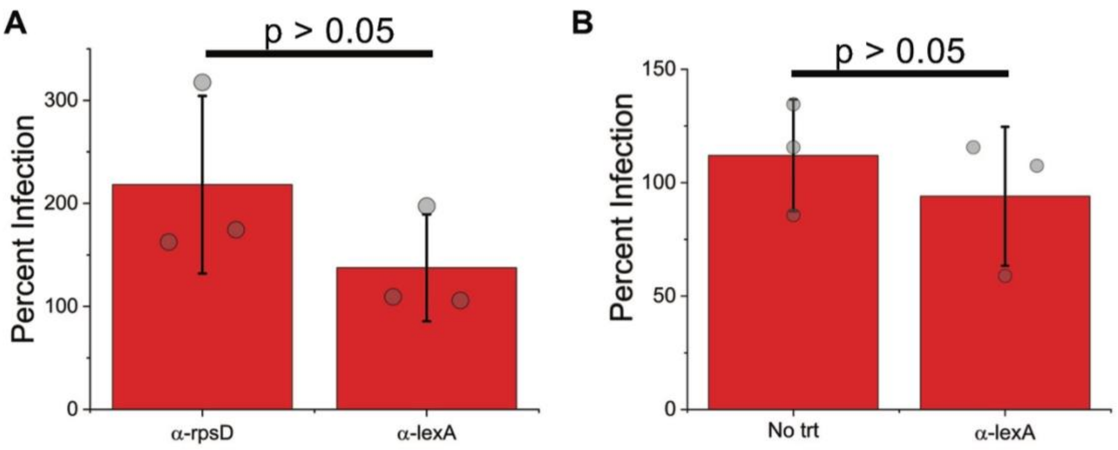

Figure S18. Effect of 45-minute incubation with PNA treatment during infection. (**A**) Individual colonies of SL1344 were picked off of solid media and grown for 16 hours in LB medium supplemented with 30 µg/mL streptomycin, diluted 1:10, and regrown for 4 hours. After regrowth bacteria was diluted to a concentration equivalent to a 30 MOI of 200,000 cells/mL in 100 µL (conditions of HeLa cell infection after 24 hours growth). Cultures were suspended in phosphate buffered saline and treated with 10 µM of either α-lexA, α-rpsD, or PSB for 45 minutes before serial dilution, plated on solid media (40 µg/mL streptomycin), and incubated for 16 hours. Colony forming units were counted to enumerate percent infection with respect to no treatment. There is no significant difference ($p>0.05$) between α-lexA and α-rpsD at 45 minutes of treatment. (**B**) Cultures were grown and treated as described for panel A except that prior to treatment an aliquot was removed from each biological replicate as a no treatment condition for that biological replicate. Percent infection was then calculated in comparison to each replicates' no treatment. Error bars are standard deviation for three biological replicates. Grey circles indicate individual biological replicates.

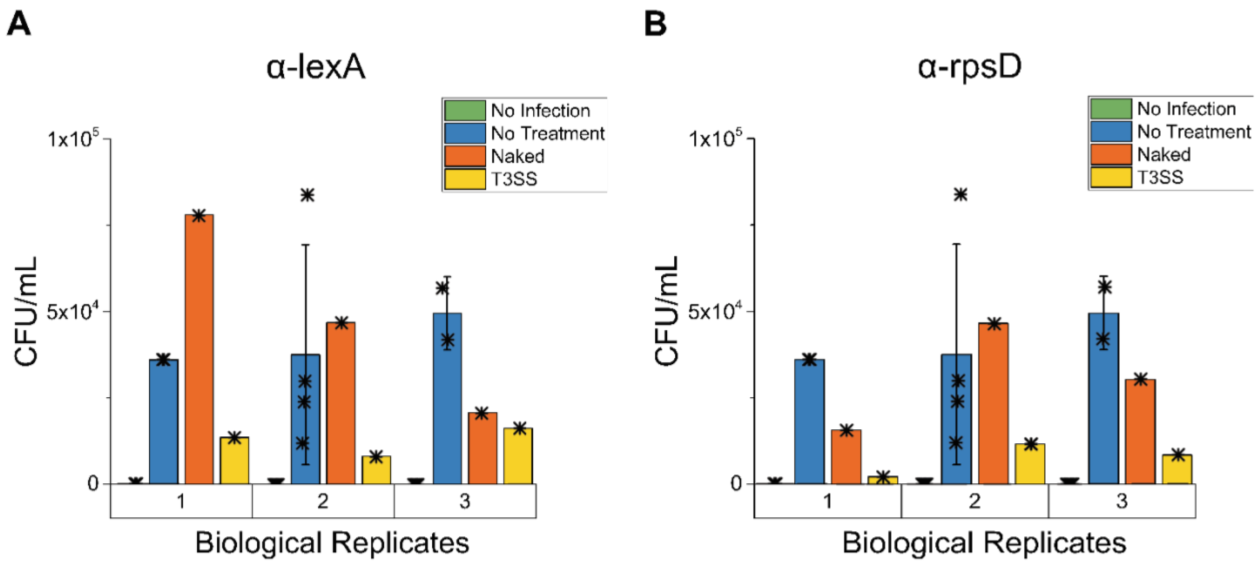

Figure S19. Raw CFU/mL of HeLa infection treated by T3SS. Intracellular STm-GFP (SL1344) colonies were measured by lysing infection HeLa cells with 30 µL of 0.1% Triton for 15 minutes at room temperature, followed by 1:10 dilution by addition of 270 µL DPBS. The lysate was serially diluted 10X, and 10 µL were plated onto LB agar with 40 µg/mL of Streptomycin. Following overnight growth colonies were counted to determine the CFU/mL. Shown are individual HeLa biological replicates shown in Figure. 4C where error bars are standard deviation between technical replicates shown as asterisks.

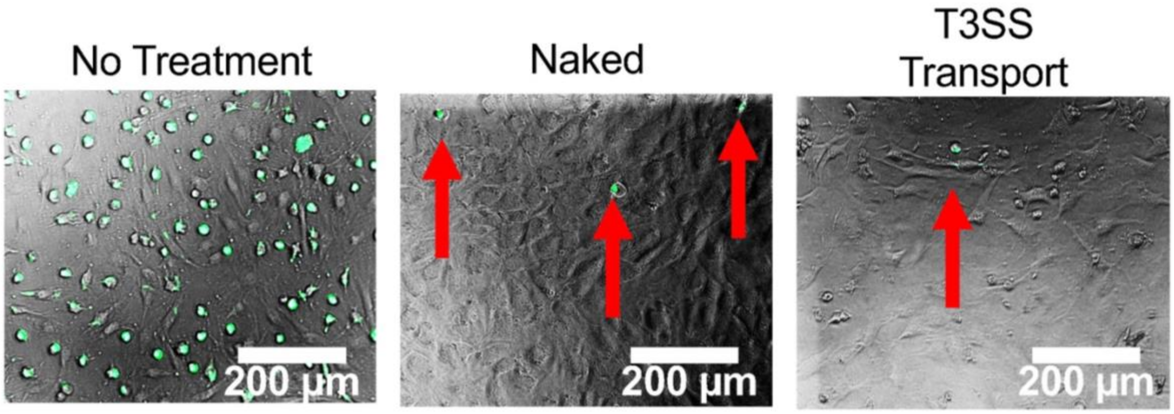

Figure S20. **T3SS-PNA treatment eliminates *Salmonella* infection of osteoblast cells**. Osteoblast precursor cells were grown for 24 hours on tissue culture treated 96-well plates prior to infection with STm- GFP (SL1344) at a multiplicity of infection of 30. The antisense inhibitor α-rpsD was added at 10 µM either without infection (No Treatment), after infection (Naked), or during infection (T3SS-PNA). After treatment for 18 hours the infection was imaged in brightfield and GFP channel. GFP expression corresponds to an intracellular infection with red arrows indicating hard to see GFP expression.

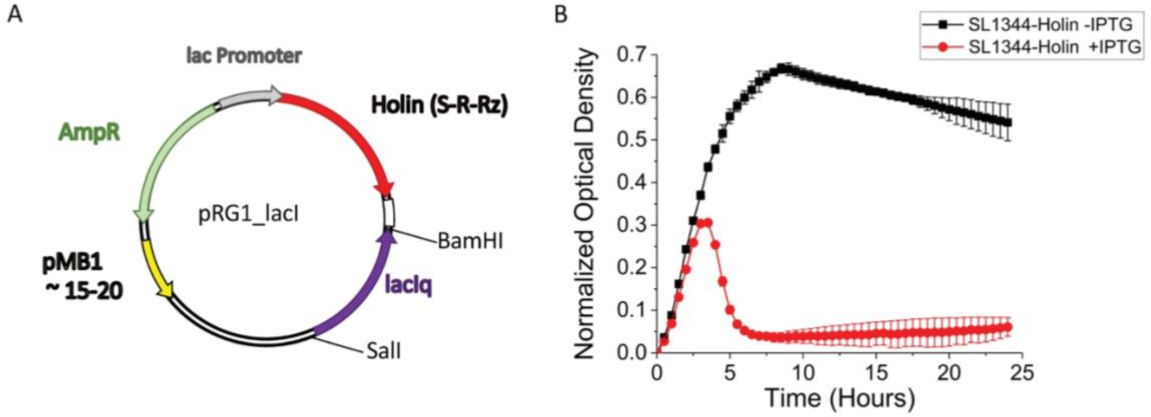

Figure S21. Plasmid map and growth curves of IPTG induced lysis of SL1344-Holin. (**A**) Plasmid map of pRG1 modified to include the lacIq gene showing the Holin-Endolysin cassette, Ampicillin resistance gene, lac promoter, and pMB1 origin of replication. (**B**) Optical density of SL1344-Holin (Delivery STm) with or without 1 mM IPTG was measured at 600 nm and normalized optical density is reported as absorbance at 600 nm minus media blank and each biological replicate normalized to the optical density at time zero. 1 mM IPTG was added for induction. Error bars indicate standard deviation of n=3 biological replicates.

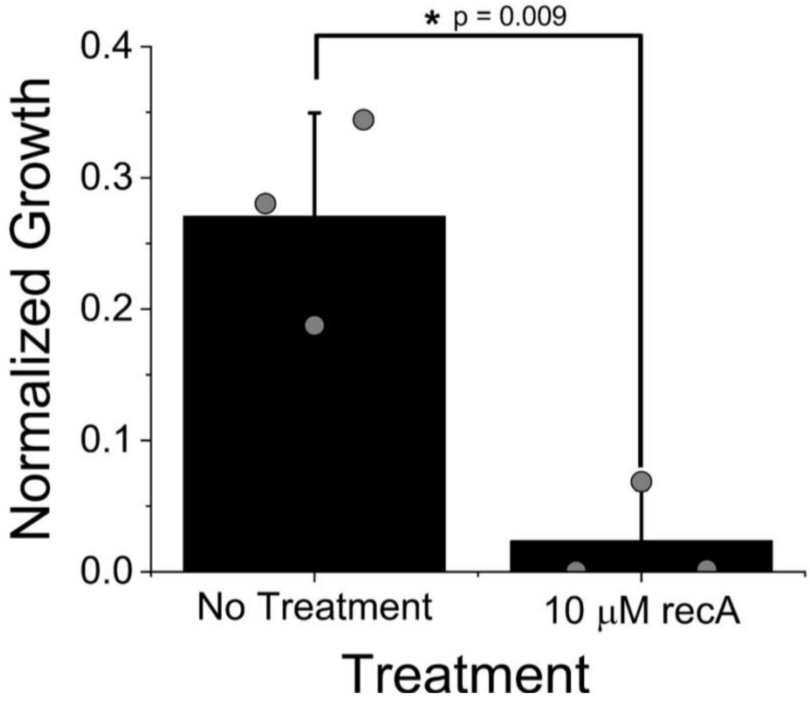

Figure S22. α-recA growth inhibition of SL1344. Optical density was measured at 600 nm in 50 μL cultures and normalized optical density is reported at 24 hours as absorbance at 600 nm minus media blank and each biological replicate normalized to the optical density reading at time zero. Overnight cultures of SL1344 were diluted 1:10,000 and treated with10 μM addition of α-recA for 24 hours. Error bars indicate standard deviation of n=3 biological replicates. Grey circles indicate individual biological replicates.

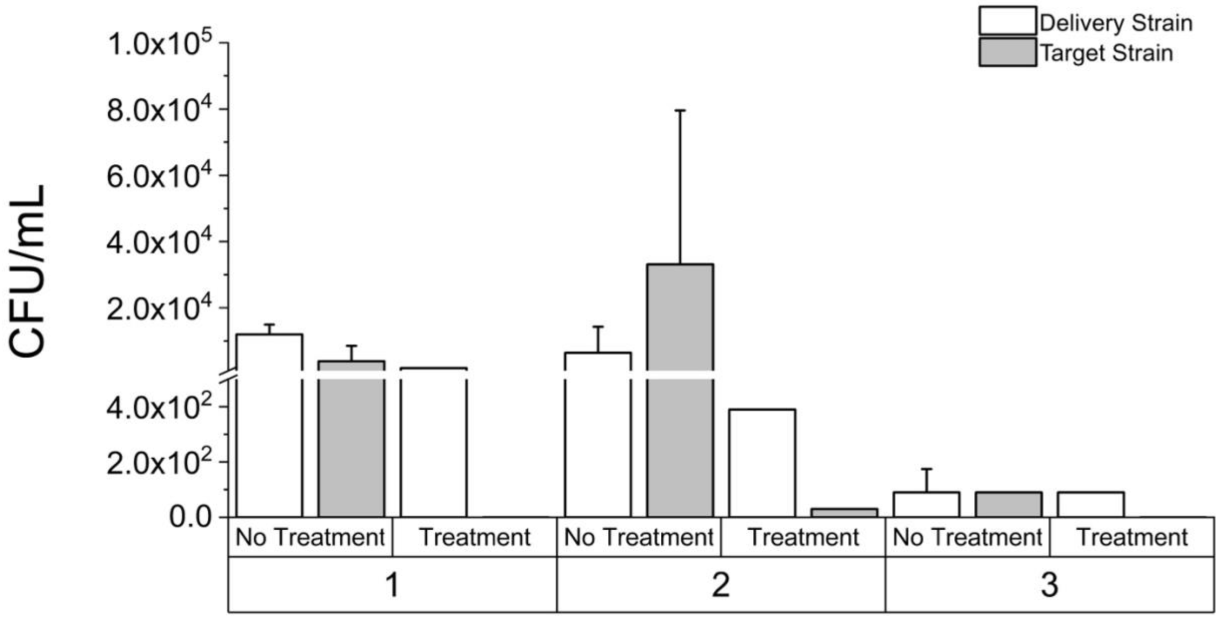

Figure S23. Raw CFU/mL in double infection study. Following double infection experiments colony forming units were counted for both Target STm (SL1344-mCherry, determined by florescence) and Delivery STm (SL1344-Holin) (Fig. 4E). Intracellular SL1344 colonies were enumerated by lysing with 30 µL of 0.1% Triton for 15 minutes at room temperature then diluted 1:10 by addition of 270 µL of DPBS. The lysate was serially diluted 1:10 in 100 µL and 10 µL plated onto LB agar with 40 µg/mL of Streptomycin and 100 µg/mL Ampicillin. Error bars indicate standard deviation of technical replicates. The biological replicates are indicated by number 1,2, and 3 on x-axis.

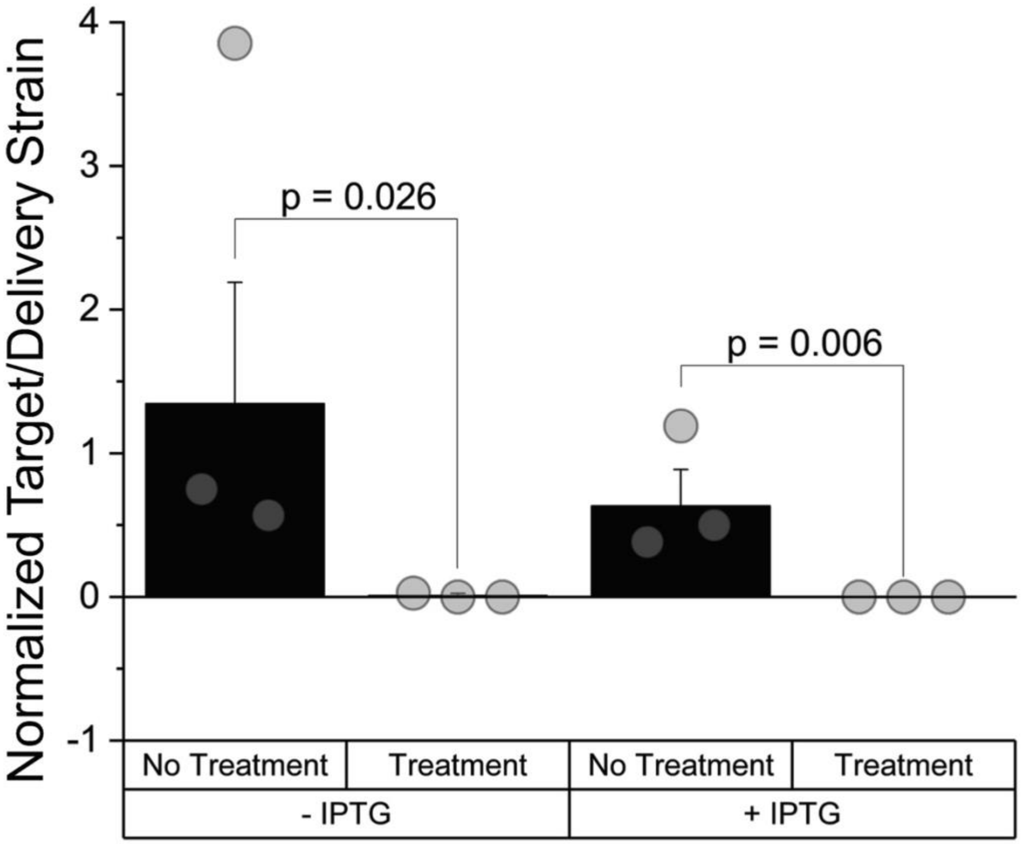

Figure S24. Normalized Target STm to Delivery STm treatment with IPTG induction of PLac promoter. The double infection clearance described in Fig. 4D but in presence of 1 mM IPTG to induce pLac promoter controlling expression Holin/Endolysin gene in Delivery STm to release PNA (α-recA). Significant decrease in the normalized Target STm to Delivery STm was measured in both absence (-IPTG, $p=0.026$) and presence of IPTG (+IPTG, $p=0.006$). This data showed that the leaky expression (-IPTG) was sufficient to turn on Holin/Endolysin based kill switch. Error bars indicate standard deviation of $n=3$ biological replicates. Grey circles indicate individual biological replicates.

Supplementary Tables

Table S1. CLSI sensitive/resistant breakpoints. CLSI breakpoints (µg/mL) for 2016-2017[5] were used to determine antibiotic resistance of clinical isolates.

Antibiotic	Sensitive	Intermediate	Resistant
Ampicillin (AMP)	8	16	32
Ceftriaxone (FRX)	1	2	4
Meropenem (MER)	1	2	4
Gentamicin (GEN)	4	8	16
Kanamycin (KAN)	16	32	64
Tetracycline (TET)	4	8	16
Ciprofloxacin (CIP) (*E. coli* and *K. pneumoniae*)	1	2	4
Ciprofloxacin (*Salmonella enterica*)	0.06	0.125	1
Nalidixic Acid (NXA)	6	N/A	32
Chloramphenicol (CHL)	8	16	32

Table S2. MDR Clinical Isolate Antibiotic MIC90. Antibiotic concentration (in µg/mL) ranges at which 90% of growth inhibition occurs at 24 hours are indicated for each bacterial clinical isolate.

	**CRE E. coli**	**MDR E. coli**	**ESBL KPN**	**NDM-1 KPN**	**MDR STm**
**CHL**	>32	<8	>32	8-16	<8
**NXA**	>32	>32	>32	>32	<6
**CIP**	>4	>4	>4	>4	<1
**TET**	>16	4-8	>16	>16	8-16
**KAN**	>64	<16	>64	>64	<16
**GEN**	>16	<4	<4	>16	<4
**MER**	4	2-4	>4	>4	<1
**FRX**	>4	>4	>4	>4	>4
**AMP**	>32	>32	>32	>32	>32

Table S3. Unique antibiotic resistance genes identified in clinical isolates. Italicized label represents antibiotic class the gene confers resistance to where: *Bla* is for β-lactam resistance, *Flq* is for fluoroquinolone resistance, *AGly* is for aminoglycoside resistance, *Phe* is for phenicol resistance, *Tet* is for tetracycline resistance, *Sul* is for sulfonamide resistance, and *Tmt* if for trimethoprim resistance. Non-italicized portion is the unique gene identified by ARG-ANNOT.

**CRE *E. coli***	**MDR *E. coli***	**ESBL KPN**	**NDM-1 KPN**	**STm**
Bla AmpC1	*Bla* AmpC2	*Bla* SHV-11	*Bla* TEM-217	*AGly* Aac6-Iaa
Bla AmpH	*Bla* PBP	*Bla* AmpH	*Bla* NDM-1	*Bla* PBP
Bla AmpC2	*Bla* ampH	*Bla* Oxa-9	*Bla* CTX-M	
Bla CMY-94	*Bla* TEM-219	*Bla* TEM-171	*Bla* SHV-73	
Bla PBP	*Bla* TEM-10	*Bla* TEM-220	*Bla* PBP	
AGly AadB	*Bla* CTX-M	*Bla* KPC-3	*Bla* AmpH	
AGly StrA/B	*AGly* Sat-2A	*Bla* PBP	*Bla* PBP	
Phe PheCml45	*Tmt* DfrA1	*Flq* OqxBgb	*Flq* QnrB1	
Tet TetB	*Phe* CatA1	*AGly* AadA1-pm	*Flq* Qnr-S1	
Sul SulI		*AGly* Aac6-Ib	*Flq* OqxBgb	
Tmt Dfr24			*AGly* StrB	
			AGly RmtF	
			AGly Ant3	
			Tet TetA/R	
			Sul SulI	
			Tmt DfrA1	

Table S4. Genetic and phenotypic antibiotic resistance characterization. Pink cells correspond to full resistance across an antibiotic class. Yellow is partial resistance to the class, and green is full sensitivity to the class.

	**CRE *E. coli***	**MDR *E. coli***	**ESBL KPN**	**NDM-1 KPN**	**STm**
**Antibiotic Type**	**Number of Resistance Genes**				
β-lactams	5	6	7	7	1
Aminoglycosides	2	1	2	3	1
Tetracyclines	1	0	0	1	0
Fluorquinolones	0	0	1	3	0
Phenicols	1	1	0	0	0

Table S5. Predicted 0-bp mismatch off-targets of antisense-PNA molecules in each reference bacterial genome and clinical isolate. The label “STC” denotes gene off-targets for which the antisense-PNA aligns to the start codon, and which will therefore be much more likely to experience translational inhibition.

**Bacterial Reference Genome Results**			
**PNA**	***E. coli* MG1655**	***K. pneumoniae* MGH 78578**	***Salmonella enterica* serovar Typhimurium SL1344**
**α-folC**	None	KPN_04193 (putative 6-phosphofructokinase)<br>*uxaC*	None
**α-rpsD**	*cueO*<br>*narI*	Non-protein coding region	*wcaM*<br>*rtcA* (STC)
**α-ffh**	None	None	None
**α-lexA**	None	None	None
**α-gyrB**	Last 5 nt of *psiE*	None	None
**α-acrA**	*narX* (STC)	MFS transporter (STC), non-protein coding region	None
**α-csgD**	Non-protein coding region	Non-protein coding region	Non-protein coding region (2)
**α-fnr**	None	None	FNR (non STC)
**α-recA**	None	None	None

**Clinical Isolates Results**					
	**CRE *E. coli***	**MDR *E. coli***	**ESBL KPN**	**NDM-1 KPN**	**STm**
**α-folC**	Prokka 00542<br>Prokka 03005	None	*pfka1*	*pfkA1*	None
**α-rpsD**	*cueO*	*cueO*	*frlD*<br>Non-protein coding region	Non-protein coding region	Prokka03791<br>Prokka 03488<br>*rtcA* (STC)
**α-ffh**	*uhpC*	*uhpC*	*yhes1*	*yheS2*	None
**α-lexA**	None	None	None	None	None
**α-gyrB**	Non-protein coding region<br>Last 5 bp of *yhbX*	None	Non-protein coding region	None	None
**α-acrA**	*narX* (STC), *livH*	*narX* (STC), *livH*	*exuT* (STC)	*exuT* (STC)	None

**α-csgD**	None	Sodium:sulfate symporter	Hypothetical protein	None	None
**α-fnr**	None	None	None	None	None
**α-recA**	None	None	None	None	None

Table S6. DNA oligonucleotides containing PNA-target gene sequence that were utilized for DNA-PNA complex formation in Electrophoretic Mobility Shift Assay. Synthetic DNA oligonucleotides were 60 nt in length (purchased from IDT), contained the PNA-target gene sequence bolded and underlined. The nonsense oligonucleotide was a randomized 60nt sequence that had no complementarity for any PNA designed for the study served as a non-binding control.

Oligo/Primer Purpose	Target gene	Oligo/Primer Sequence (5' to 3')
Antisense oligomer for α-rpsD	*rpsD*	ATT TAG GTG ACA CTA TAG AAG TGG AGA A**AG AAA *ATG* GCA A**GA TAT TTG GGT CCT AAG CTC
Antisense oligomer for α-lexA	*lexA*	ATT TAG GTG ACA CTA TAG AAG CAG GGG G**CG GA*A* *TG*A AAG C**GT TAA CGG CCA GGC AAC AAG
Nonsense oligomer	N/A	GAA TTC GAA TTC GGT CAG TGC GTC CTG CTG ATG TGC TCA GTA TCT CTA TCA CTG ATA GGG

Supplementary References

1. Das, U. & Shuman, S. 2'-Phosphate cyclase activity of RtcA: a potential rationale for the operon organization of RtcA with an RNA repair ligase RtcB in Escherichia coli and other bacterial taxa. *RNA* **19**, (2013) doi:10.1261/rna.039917.113.
2. Jensen, K. K., Ørum, H., Nielsen, P. E. & Nordén, B. Kinetics for Hybridization of Peptide Nucleic Acids (PNA) with DNA and RNA Studied with the BIAcore Technique. *Biochemistry* **36**, (1997) doi:10.1021/bi9627525.
3. Gruber, A. R., Lorenz, R., Bernhart, S. H., Neuböck, R. & Hofacker, I. L. The Vienna RNA websuite. *Nucleic Acids Res.* **36**, (2008) doi:10.1093/nar/gkn188.
4. Szklarczyk, D. *et al.* STRING v10: Protein-protein interaction networks, integrated over the tree of life. *Nucleic Acids Res.* **43**, (2015) doi:10.1093/nar/gku1003.
5. CLSI. Performance Standards for Antimicrobial Susceptibility Testing. *CLSI Suppl. M100* (2017).
