# Supplementary material for: Facile accelerated specific therapeutic (FAST) platform develops antisense therapies to counter multidrug-resistant bacteria
Source: Commun Biol. 2021 Mar 12;4:331. doi: 10.1038/s42003-021-01856-1 (PMC7955031; doi:10.1038/s42003-021-01856-1)
Supplement: Supplementary file 5 — Reporting Summary [file 42003_2021_1856_MOESM5_ESM.pdf]

nature research

Corresponding author(s): COMMSBIO-19-1859A

Last updated by author(s): Jan 23, 2021

Reporting Summary

Nature Research wishes to improve the reproducibility of the work that we publish. This form provides structure for consistency and transparency in reporting. For further information on Nature Research policies, see our Editorial Policies and the Editorial Policy Checklist.

Statistics

For all statistical analyses, confirm that the following items are present in the figure legend, table legend, main text, or Methods section.

n/a	Confirmed	
☐	☒	The exact sample size (*n*) for each experimental group/condition, given as a discrete number and unit of measurement
☐	☒	A statement on whether measurements were taken from distinct samples or whether the same sample was measured repeatedly
☐	☒	The statistical test(s) used AND whether they are one- or two-sided<br>*Only common tests should be described solely by name; describe more complex techniques in the Methods section.*
☐	☒	A description of all covariates tested
☒	☐	A description of any assumptions or corrections, such as tests of normality and adjustment for multiple comparisons
☐	☒	A full description of the statistical parameters including central tendency (e.g. means) or other basic estimates (e.g. regression coefficient) AND variation (e.g. standard deviation) or associated estimates of uncertainty (e.g. confidence intervals)
☐	☒	For null hypothesis testing, the test statistic (e.g. *F*, *t*, *r*) with confidence intervals, effect sizes, degrees of freedom and *P* value noted<br>*Give P values as exact values whenever suitable.*
☒	☐	For Bayesian analysis, information on the choice of priors and Markov chain Monte Carlo settings
☒	☐	For hierarchical and complex designs, identification of the appropriate level for tests and full reporting of outcomes
☐	☒	Estimates of effect sizes (e.g. Cohen's *d*, Pearson's *r*), indicating how they were calculated

Our web collection on statistics for biologists contains articles on many of the points above.

Software and code

Policy information about availability of computer code

Data collection	No software was used
Data analysis	The PNA Finder toolbox is built using Python 3.7, Bowtie 2 (version 2.3.5.1), the read alignment processing program SAMtools (1.9), and the feature analysis program BEDTools (v2.25.0). All custom scripts have been made available at https://github.com/taunins/pna_finder. Determination of Pearson’s coefficients and their p-values, in Figures 3A and 3N, were done using the statistical software JMP Pro 15; all other statistical tests were done in Excel.

For manuscripts utilizing custom algorithms or software that are central to the research but not yet described in published literature, software must be made available to editors and reviewers. We strongly encourage code deposition in a community repository (e.g. GitHub). See the Nature Research guidelines for submitting code & software for further information.

Data

Policy information about availability of data

All manuscripts must include a data availability statement. This statement should provide the following information, where applicable:

- Accession codes, unique identifiers, or web links for publicly available datasets
- A list of figures that have associated raw data
- A description of any restrictions on data availability

All data needed to evaluate the conclusions in the paper are present in the paper and/or the Supplementary Materials. Additional data available from authors upon request. Sequence data for the MDR clinical isolates used to identify antibiotic resistance genes and analyze PNA homology have been deposited in GenBank with the accession codes WWEV00000000.1, MSDR00000000.1, WWEX00000000.1, WWEY00000000.1, and WWEW00000000.1 for MDR E. coli, CRE E. coli, ESBL KPN, NDM-1 KPN, and MDR STm respectively.

Field-specific reporting

Please select the one below that is the best fit for your research. If you are not sure, read the appropriate sections before making your selection.

☒ Life sciences ☐ Behavioural & social sciences ☐ Ecological, evolutionary & environmental sciences

For a reference copy of the document with all sections, see nature.com/documents/nr-reporting-summary-flat.pdf

Life sciences study design

All studies must disclose on these points even when the disclosure is negative.

Sample size	A minimum of three biological replicates were used in determining growth rates and growth curves, with three biological replicate no treatments controls always run along side, as this is the standard in the field.
Data exclusions	No data was excluded.
Replication	Clinical isolate antibiotic sensitivity was replicated when doing PNA and antibiotic combination experiments
Randomization	Sample randomization was not required for this study as no bias in experimental groups is expected.
Blinding	No blinding was performed in this study.

Reporting for specific materials, systems and methods

We require information from authors about some types of materials, experimental systems and methods used in many studies. Here, indicate whether each material, system or method listed is relevant to your study. If you are not sure if a list item applies to your research, read the appropriate section before selecting a response.

Materials & experimental systems

n/a	Involved in the study	
☒	☐	Antibodies
☐	☒	Eukaryotic cell lines
☒	☐	Palaeontology and archaeology
☒	☐	Animals and other organisms
☒	☐	Human research participants
☒	☐	Clinical data
☒	☐	Dual use research of concern

Methods

n/a	Involved in the study	
☒	☐	ChIP-seq
☒	☐	Flow cytometry
☒	☐	MRI-based neuroimaging

Eukaryotic cell lines

Policy information about cell lines

Cell line source(s)	HeLa, MC3T3-E1, and RAW264.7 are all from ATCC
Authentication	None of the cell lines used were authenticated
Mycoplasma contamination	The cell lines were not tested for mycoplasma contamination.
Commonly misidentified lines (See ICLAC register)	No commonly misidentified cell lines were used in this study.
